# Physiological and metabolic analyses reveal the proline-mediated flowering delay mechanism in *Prunus persica*

**DOI:** 10.3389/fpls.2024.1302975

**Published:** 2024-04-25

**Authors:** Yeonju Park, Pandiyan Muthuramalingam, Jae Hoon Jeong, Seung Heui Kim, Hyunsuk Shin

**Affiliations:** ^1^ Department of GreenBio Science, Gyeongsang National University, Jinju, Republic of Korea; ^2^ Division of Horticultural Science, Gyeongsang National University, Jinju, Republic of Korea; ^3^ Fruit Research Division, National Institute of Horticultural and Herbal Science, Wanju, Republic of Korea; ^4^ Department of Fruit Science, Korea National College of Agriculture and Fisheries, Jeonju, Republic of Korea

**Keywords:** 5AG, computational metabolomics, flowering delay, proline pathway, *Prunus persica*, qRT-PCR

## Abstract

Peaches are susceptible to various environmental stresses. Particularly in late spring, freezing temperatures can damage peaches and consequently, affect their productivity. Therefore, flowering delay is a prominent strategy for avoiding spring frost damage. Our previous study confirmed that treatment with 5% sodium alginate and 100 mM CaCl_2_ (5AG) to avoid frost damage during the blooming stage delays flowering. To reveal the flowering delay mechanism of peaches, this study systematically analyzed the modification of amino acid profiles in control and 5AG-treated peach plants at different day intervals. Our findings indicate that arginine (Arg), glutamate (Glu), and proline (Pro) levels differed between the control and 5AG-treated peach shoots throughout the phenological development of flower buds. Furthermore, two amino acids (Arg and Glu) are involved in the Pro pathway. Thus, using a computational metabolomics method, Pro biosynthesis and its characteristics, gene ontology, gene synteny, *cis*-regulatory elements, and gene organizations were examined to decipher the involvement of Pro metabolism in peach flowering delay. In addition, qRT-PCR analysis revealed the transcriptional regulation of Pro-related and flowering-responsive genes and their role in flowering delay. Overall, this pilot study provides new insights into the role of Pro in the flowering delay mechanisms in *Prunus persica* through 5AG treatment.

## 1 Introduction

Plants are sessile, and hence cannot escape from various environmental conditions that pose a major hurdle throughout their life cycle (Muthuramalingam et al., 2017). Under environmental conditions, abiotic stresses such as cold, freezing and heat (extreme temperatures), drought, and salinity are the main factors that can create unfavorable conditions for plant growth and development. Furthermore, these stressors influence the geographical distribution of plants and reduce agricultural productivity (Krasensky and Jonak, 2012).

Cold conditions, including freezing, are among the major abiotic stressors encountered by deciduous fruit trees, particularly peach (*Prunus persica*) trees. Peach is a highly prized, important seasonal fruit grown worldwide, including in South Korea (Muthuramalingam et al., 2022). Over the past decade, owing to climate change and global warming, the peach blooming period has advanced by approximately 13 days (https://data.kma.go.kr). The peach growing regions and production in southern South Korea have frequently suffered from damage caused by increased average temperatures (2°C–4°C) in February and March (early spring), followed by sudden frost events in late spring. Woody fruit trees such as peaches acquire freezing tolerance (FT) via cold acclimation and lose FT through deacclimation (DA) in response to rising temperatures in order to survive winter and resume growth in the spring. However, owing to recent climate change, peach flowers are forced to bloom earlier through premature DA, which increases the risk of frost damage to peach flowers or buds. Therefore, there is a need for a chemical treatment strategy that can delay the blooming date and budburst of peaches, thereby avoiding the risk of frost injury (Park and Shin, 2022).

Several studies have investigated blooming delays in deciduous temperate fruit trees using various materials and treatment time intervals to avoid spring frost injury (Crisosto et al., 1990; Myers et al., 1996; Samani et al., 2005; Deyton et al., 2009; Mohammadi et al., 2015). In our previous study, we found that a combination of sodium alginate and CaCl_2_ significantly delayed peach blooming by up to 9 days (Park and Shin, 2022). Ion exchange that takes place between Na^+^ in sodium alginate and Ca^2+^ in CaCl_2_ is the primary mechanism of encapsulation by AG treatment (Rihan et al., 2017). When Ca^2+^ participates in the ionic exchange binding between G-blocks in the sodium alginate polymer chain, it creates a three-dimensional network (Rezende et al., 2007). Based on this principle, AG treatment can slow down the flowering process by forming a physical barrier on the surface of the peach flower buds.

In general, amino acids can act as precursors for the biosynthesis of signaling molecules and secondary metabolites. Among these amino acids, proline (Pro) is an efficient signaling molecule, whereas others are precursors for synthesizing secondary metabolites and plant hormones. Moreover, Pro can be involved in chemical defenses against both biotic and abiotic stresses (Szabados and Savouré, 2010; Hildebrandt et al., 2015; Shin et al., 2016). Under stress conditions, Pro biosynthesis is upregulated, whereas Pro catabolism is activated in the dark and during stress relief (Szabados and Savouré, 2010). Metabolic modifications of Pro, such as osmolytes, ROS scavengers, and protein chaperones, can reduce cell damage (Rizzi et al., 2015). Pro is also involved in various developmental processes such as growth, cell division, differentiation, flowering, and embryo development (Szabados and Savouré, 2010; Kavi Kishor et al., 2015; Shin et al., 2016; Wei et al., 2022). In particular, Pro accumulation has been observed in tomato plants exposed to warm temperatures during flowering (Schwacke et al., 1999). Furthermore, our previous studies demonstrated that Pro accumulation appeared in peaches as a physiological reaction related to spring growth resumption (Shin et al., 2016; Oh et al., 2017).

Keeping this importance and lacunae in mind, in this study, peach floral buds were coated with a mixture of sodium alginate and CaCl_2_, and the subsequent changes in amino acid profiles were assessed. Additionally, a computational metabolomics approach was employed to identify the involvement of Pro related genes in peaches. This is the first holistic study to combine phenological and computational metabolomic analyses to elucidate the relationship between the flowering delay and amino acid level changes in peach plants. Specifically, this study aimed to determine whether 5AG, a frost avoidance strategy, induces delayed flowering through physiological and metabolic changes in *P. persica*. Furthermore, this study sought to identify the mechanisms underlying flowering delay related to Pro metabolism by elucidating the molecular, physiological, and physiochemical roles of Pro.

## 2 Materials and methods

The overall framework of 5AG treatment followed by amino acid profiling and analysis of the Pro pathway-associated genes through integrated physiological and computational metabolomic analyses is illustrated in **Supplementary Figure 1**.

### 2.1 Plant materials and treatment

In this study, ‘Kawanakajima Hakuto’ peach trees grafted on rootstocks of *P. persica*, which is one of the major peach cultivars in South Korea and is commonly used as a reference variety for mid-cold tolerance cultivars under cultivation conditions (Yun et al., 2014), were selected as a plant material. Six-year-old peach cultivar ‘Kawanakajima Hakuto’ shoots were used from a conventional peach orchard (35^°^07’50.8’’N, 128^°^09’46.7’’E), Jinju, South Korea. The chosen peach shoots were then treated with 5% sodium alginate and 100 mM CaCl_2_ (5AG), except for CT shoots, which were treated with distilled water. The majority of peach flower buds reached the 2nd stage of phenological flower development in the experimental orchard. Eight shoots from six healthy peach trees were randomly selected for the treatment on 8 March 2021. We sprayed the 5AG treatment solution onto selected shoots with a compressed sprayer until the flower buds were completely coated. For this 5AG treatment, 100 mM CaCl_2_ was initially applied to flower buds, followed by sodium alginate to form a better gel complexation/encapsulation, in accordance with the results of our previous experimental report (Park and Shin, 2022). After treatment, all flower buds were checked to confirm that they were properly coated with 5AG.

### 2.2 Amino acid extraction and profiling

The phenological stages of peach flowers were divided into five stages: 2nd stage (swollen bud), 3rd stage (calyx green), 4th stage (calyx red), 5th stage (first pink), and 6th stage (first bloom) (**Supplementary Figure 2**). Peach shoots, approximately 30 cm in length, were collected each DAT (days after treatment). The sampling dates were selected based on the expression rate of CT in each phenological stage of the peach flower: 0 (basic condition), 1 to 2 (immediately after treatment), 11 (more than 50% of stage 3), 21 (after maximum expression of stage 4), 25 (after maximum expression of stage 5), and 28 DAT (more than 80% of stage 6, full bloom). These samples were immediately ground in liquid nitrogen using a TissueLyser II (Qiagen) and stored as powder at −80°C until further use. Amino acid extraction was conducted with minor modifications based on the method of Shin et al. (2018). Tubes containing 2 g of the sample from each DAT were added to 30 mL of 1% sulfosalicylic acid and vortexed. Subsequently, the samples were centrifuged twice (at 1,350×*g* for 10 min and at 7,830×*g* for 20 min). The supernatant was then filtered using a 0.2 µm nylon syringe filter. The extracted free amino acid samples were quantified using a Hitachi amino acid analyzer (Hitachi L-8900, Tokyo, Japan) equipped with an HPLC packed column. A series of Kanto L-8900 buffers, PF-1-4 and RG were used as the mobile phase. All the samples were individually injected and quantified using a ninhydrin coloring solution kit (Wako Chemicals Inc., Osaka, Japan). The amino acid content levels were then visualized as a heatmap using Multi Experiment Viewer (MeV) v4.9 (Saeed et al., 2003).

### 2.3 Pathway analysis and identification of gene features

The flower development-linked amino acid/metabolite, proline (Pro) was subjected to Genome Database for Rosaceae (GDRCyc; https://ptools.rosaceae.org/) database, and Pro biosynthesis pathways and Pro-related genes were identified. The gene details were then imported into the GDR gene search tool (https://www.rosaceae.org/search/genes) to obtain the chromosome number, genomic and coding sequences, amino acid length, molecular weight, and isoelectric point details.

### 2.4 Gene structure analysis

Pro biosynthesis pathway-associated genes and their genomic and coding sequences were analyzed using the GSDS online tool v2.0 (Hu et al., 2015) to impute the arrangements of upstream and downstream regions, introns, and exons.

### 2.5 Promoter analysis

Pro metabolism-related genes with their encoding sequences were loaded onto the plant *cis*-acting regulatory DNA element (PLACE) web server (https://www.dna.affrc.go.jp/PLACE; Higo et al., 1999) to predict the *cis*-regulatory elements. All possible *cis*-regulatory elements present in the promoter region of Pro-related genes were matched in the 1 kb upstream of the initiation codon region of Pro-related genes using the PLACE online tool, interlinked with PLACE ID, and exactly matched with peach promoters. Promoter features, such as *cis*-element factor site name, position, and sequence details were retrieved.

### 2.6 Gene ontology analysis

Predicted Pro-related genes with their corresponding gene IDs were uploaded to the ShinyGO *v*0.741 database (http://bioinformatics.sdstate.edu/go/; Ge et al., 2020) to obtain functional GO annotation against *P. persica.* Functional GO enrichment was imputed according to the inbuilt hypergeometric test with a significant enrichment threshold level (−log10FDR) and FDR *P*-value ≤ 0.05 for the reference genes.

### 2.7 Chromosomal collinearity

Pro-related genes and their orthologous genes in *Pyrus communis* were predicted using the inbuilt species comparison operation tool in GDRCyc (https://ptools.rosaceae.org/). Gene syntenic relationships were observed using Circos v0.55 (http://circos.ca/; Krzywinski et al., 2009).

### 2.8 RNA extraction and qRT-PCR analysis

The frozen shoot samples were ground in liquid nitrogen using TissueLyser II (Qiagen), and total RNA was isolated using the RNeasy® Plant Mini Kit (Qiagen, Valencia, CA, USA) according to the manufacturer’s protocol. The RNA sample quantity was estimated by determining the absorbance ratio of A260/A280 using a NanoPhotometer® N60/N50 spectrophotometer (Implen, TheLab Inc., USA). Afterwards, for the use of qRT-PCR, 1 µg RNA was converted into cDNA by PrimeScript™ RT reagent kit with gDNA Eraser (Perfect Real Time) (TaKaRa Biotechnology) as per the manufacturer’s instruction. qRT-PCR was performed using the Thermal Cycler Dice™ Real Time System III (TaKaRa) with TB Green® Premix Ex Taq™ (TaKaRa Biotechnology). Target reference genes and their primers are listed in **Supplementary Table 1**. Primers were designed using the GenScript Real-time PCR (TaqMan) Primer Design tool. The expression levels of the five Pro-related genes and two flowering-responsive genes were investigated under CT and 5AG treatments. *RPII* gene primer was used as an endogenous control (Oh et al., 2017). Relative fold change in gene expression (2^−^ *^ΔΔCT^*) was calculated via comparative Ct value analysis with normalization of *RPII* gene expression (Livak and Schmittgen, 2001). All qRT-PCR reactions were performed in three biological and three technical replicates, and the data were subjected to statistical analysis.

### 2.9 Statistical analysis

All experiments were performed with three biological and three technical replicates. Data were presented as mean ± standard error (SE). Significant differences between the values of the control and treated samples were analyzed through one-way analysis of variance (ANOVA), Duncan’s *post hoc* test, and a *t*-test using the SAS 9.4 software package (SAS Institute Inc., Cary, NC, USA). Data were processed and plotted using SigmaPlot 12.5 (Systat Software, Inc., San Jose, CA, USA). Asterisks (*, **, and ***) in the figures represent significant differences (*P <* 0.05, < 0.01, and < 0.001, respectively).

## 3 Results

### 3.1 Identification of flowering delay-associated amino acids

**Figure 1:**
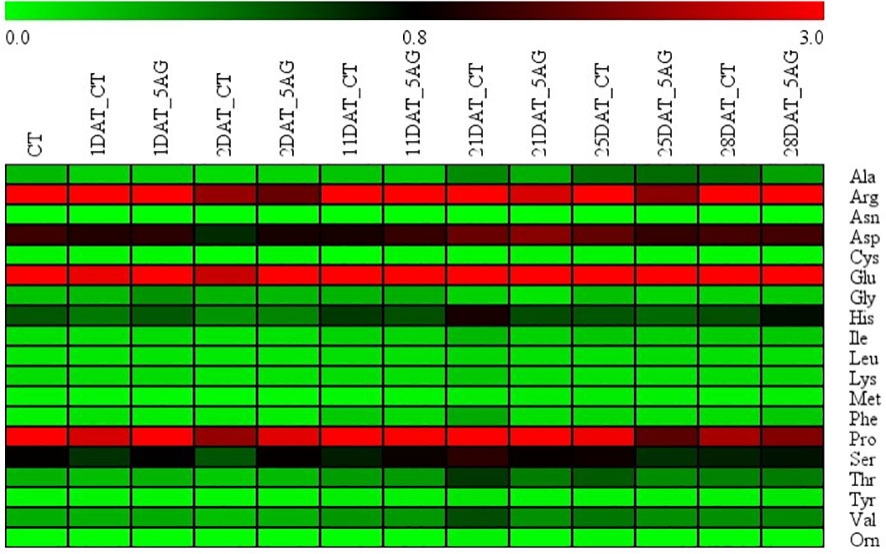
Heatmap representation of amino acid content extracted from control (CT) and 5AG-treated (5AG) peach shoot samples collected at different time intervals. Green, low; others, medium; red, high amino acid content level. The colored scale bar at the top indicates the relative amino acid content level, where 0.0, 0.8, and 3.0 represent low, medium, and high amino acid levels, respectively.

**Figure 2:**
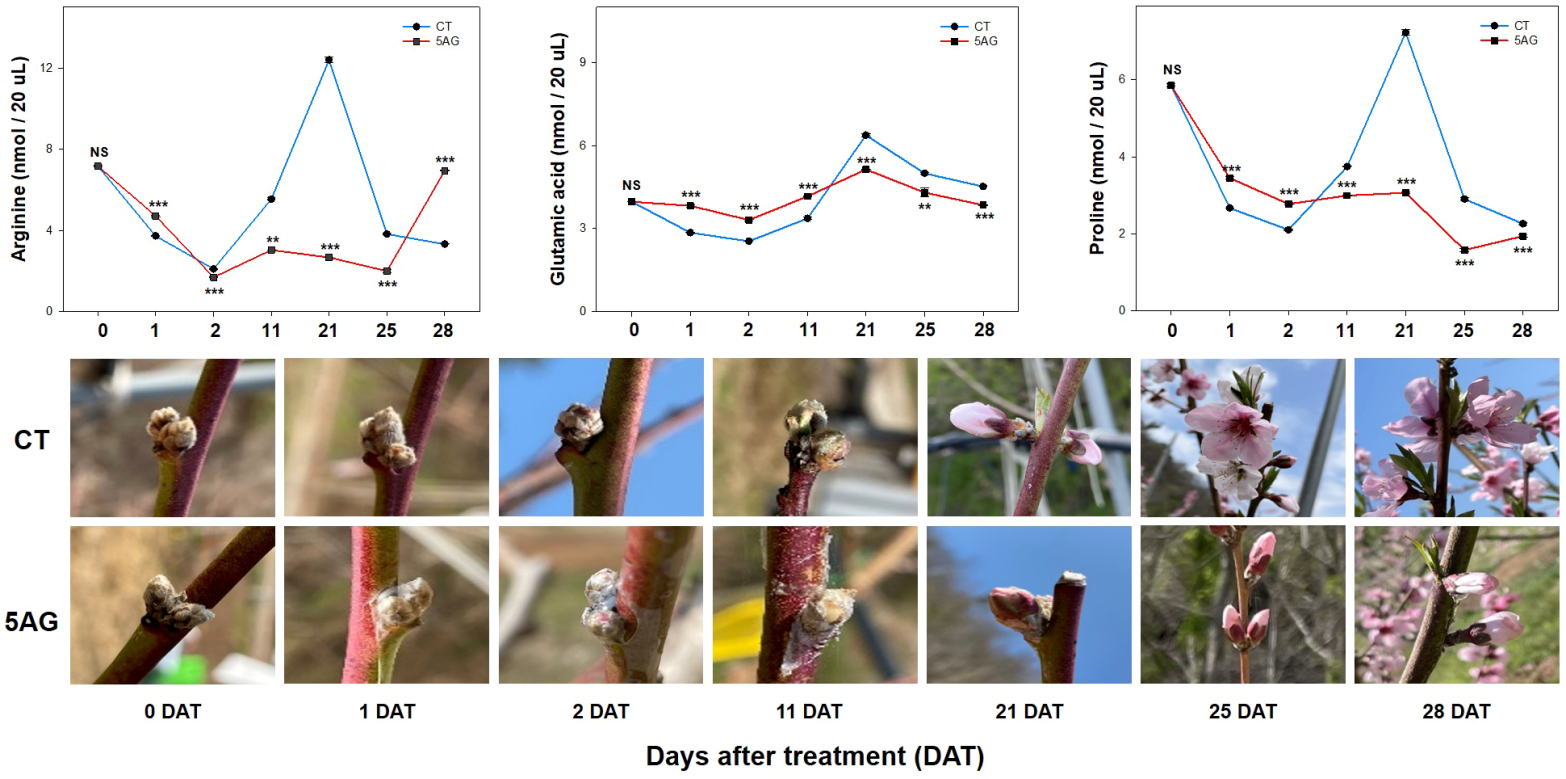
Arg, Glu, and Pro content levels were extracted from the control (CT) and 5AG-treated (5AG) peach shoot samples collected at different time intervals. Data are presented as mean ± SE (n = 3). A *t*-test was used for statistical comparisons between CT and 5AG per DAT. The presence of asterisks indicates a statistically significant difference compared to CT. **P < 0.01, ***P < 0.001, NS indicates non-significant.

Our previous study revealed that 5AG was highly effective in delaying peach flowering by approximately a week and could be a vital countermeasure to avoid frost damage (Park and Shin, 2022). Accordingly, 19 amino acids were analyzed to determine the changes in amino acid levels induced by 5AG. Arg, Glu, and Pro levels among them in CT and 5AG-treated samples varied significantly depending on the DAT, whereas the other amino acids such as Ala, Asp, Cys, Gly, His, Ile, Leu, Lys, Met, Phe, Ser, Thr, Tyr, Val, and Orn showed low-levels (**Table 1**; **Figure 1**). No change in Asn content was observed during any of the DATs in 5AG and CT (**Figure 1**). Interestingly, CT at 21 DAT had higher levels of Arg, Glu, and Pro contents as well as higher total amino acid content than 5AG (**Table 1**). Additionally, there were notable differences between CT and 5AG related to specific changes in three amino acids (Arg, Glu, and Pro) and the phenological development of peach flowers (**Figure 2**). In the initial stages (0 to 2 DAT), the levels of these three amino acids remained similar in both the CT and 5AG samples. However, in CT, the three amino acids increased drastically between 11 DAT and 21 DAT, and the development of flower buds also progressed rapidly during this period. In CT, the content of Arg, Glu, and Pro amino acids at 21 DAT reached maximum levels of 12.407, 6.375, and 7.223 nmol 20 µL^−1^, respectively, and the flower buds reached the 5th stage (first pink). Besides, Arg, Glu, and Pro amino acids in 5AG were 4.6-, 1.2-, and 2.4-fold lower, respectively, than those in CT for the same period. In addition, the phenological development of flower buds was at the 4th stage (calyx red) as the initial level, which was delayed in comparison with CT. Overall, amino acid content profiling revealed that the delay in flowering caused by AG treatment and the three amino acids were closely related. This suggests that Arg, Glu, and Pro play vital roles in peach flowering delay.

**Table 1:** Concentration (nmol 20 µL^−1^) of amino acids in control (CT) and 5AG-treated (5AG) peach samples.

Amino acid^z^	0 DAT	1 DAT		2 DAT		11 DAT		21 DAT		25 DAT		28 DAT	
	CT	CT	5AG	CT	5AG	CT	5AG	CT	5AG	CT	5AG	CT	5AG
**Ala**	0.214 ± 0.001 g^y^	0.143 ± 0.001 j	0.140 ± 0.003 jk	0.105 ± 0.003 l	0.129 ± 0.002 k	0.180 ± 0.002 h	0.160 ± 0.001 i	0.358 ± 0.001 d	0.246 ± 0.003 f	0.429 ± 0.001 c	0.462 ± 0.017 a	0.446 ± 0.006 b	0.289 ± 0.012 e
**Arg**	7.175 ± 0.015 b	3.731 ± 0.005 g	4.717 ± 0.022 e	2.107 ± 0.004 k	1.694 ± 0.005 m	5.544 ± 0.028 d	3.036 ± 0.003 i	12.407 ± 0.066 a	2.675 ± 0.004 j	3.824 ± 0.016 f	2.001 ± 0.078 h	3.324 ± 0.011 l	6.952 ± 0.018 c
**Asn**	0.000 ± 0.000 a	0.000 ± 0.000 a	0.000 ± 0.000 a	0.000 ± 0.000 a	0.000 ± 0.000 a	0.000 ± 0.000 a	0.000 ± 0.000 a	0.000 ± 0.000 a	0.000 ± 0.000 a	0.000 ± 0.000 a	0.000 ± 0.000 a	0.000 ± 0.000 a	0.000 ± 0.000 a
**Asp**	1.338 ± 0.001 e	1.117 ± 0.002 h	1.178 ± 0.006 g	0.666 ± 0.001 j	1.012 ± 0.003 i	1.008 ± 0.004 i	1.282 ± 0.001 f	1.709 ± 0.008 b	1.993 ± 0.002 a	1.662 ± 0.007 c	1.258 ± 0.047 f	1.403 ± 0.004 d	1.379 ± 0.009 d
**Cys**	0.033 ± 0.000 a	0.024 ± 0.004 a	0.017 ± 0.004 a	0.000 ± 0.000 b	0.023 ± 0.005 a	0.000 ± 0.000 b	0.022 ± 0.004 a	0.021 ± 0.009 a	0.033 ± 0.005 a	0.027 ± 0.005 a	0.028 ± 0.003 a	0.020 ± 0.005 a	0.027 ± 0.013 a
**Glu**	3.972 ± 0.002 g	2.855 ± 0.005 j	3.827 ± 0.019 h	2.545 ± 0.007 k	3.313 ± 0.011 i	3.371 ± 0.018 i	4.165 ± 0.006 f	6.375 ± 0.030 a	5.142 ± 0.011 b	5.000 ± 0.018 c	4.304 ± 0.161 e	4.518 ± 0.014 d	3.850 ± 0.018 h
**Gly**	0.183 ± 0.000 g	0.208 ± 0.001 e	0.337 ± 0.002 a	0.231 ± 0.001 c	0.219 ± 0.001 d	0.231 ± 0.001 c	0.249 ± 0.000 b	0.135 ± 0.001 i	0.073 ± 0.002 k	0.196 ± 0.001 f	0.126 ± 0.003 j	0.133 ± 0.002 I	0.158 ± 0.004 h
**His**	0.527 ± 0.000 e	0.418 ± 0.000 g	0.519 ± 0.003 e	0.335 ± 0.001 i	0.380 ± 0.001 h	0.619 ± 0.004 c	0.549 ± 0.000 d	1.030 ± 0.005 a	0.559 ± 0.001 d	0.531 ± 0.002 e	0.469 ± 0.019 f	0.549 ± 0.002 d	0.758 ± 0.005 b
**Ile**	0.094 ± 0.000 j	0.090 ± 0.000 k	0.103 ± 0.001 i	0.075 ± 0.000 l	0.090 ± 0.000 k	0.134 ± 0.001 g	0.126 ± 0.000 h	0.219 ± 0.001 a	0.138 ± 0.001 f	0.172 ± 0.001 b	0.148 ± 0.006 e	0.152 ± 0.001 d	0.167 ± 0.001 c
**Leu**	0.072 ± 0.000 h	0.070 ± 0.001 hi	0.078 ± 0.001 g	0.068 ± 0.001 i	0.071 ± 0.000 hi	0.100 ± 0.001 c	0.089 ± 0.001 f	0.125 ± 0.000 a	0.092 ± 0.001 e	0.089 ± 0.001 f	0.108 ± 0.005 b	0.095 ± 0.001 d	0.099 ± 0.002 c
**Lys**	0.102 ± 0.001 e	0.094 ± 0.000 f	0.110 ± 0.001 c	0.065 ± 0.000 i	0.080 ± 0.000 h	0.099 ± 0.001 e	0.091 ± 0.000 g	0.169 ± 0.001 a	0.095 ± 0.000 f	0.080 ± 0.000 h	0.081 ± 0.003 h	0.105 ± 0.001 d	0.122 ± 0.001 b
**Met**	0.029 ± 0.001 bc	0.019 ± 0.005 cdef	0.012 ± 0.004 ef	0.008 ± 0.000 f	0.014 ± 0.004 def	0.017 ± 0.000 cdef	0.025 ± 0.005 cde	0.053 ± 0.006 a	0.051 ± 0.008 a	0.055 ± 0.006 a	0.041 ± 0.002 ab	0.029 ± 0.004 bcd	0.046 ± 0.011 a
**Phe**	0.034 ± 0.001 j	0.084 ± 0.002 g	0.051 ± 0.000 h	0.054 ± 0.000 h	0.045 ± 0.001 i	0.165 ± 0.001 b	0.107 ± 0.000 d	0.266 ± 0.001 a	0.091 ± 0.001 f	0.116 ± 0.002 c	0.089 ± 0.003 f	0.101 ± 0.001 e	0.164 ± 0.003 b
**Pro**	5.850 ± 0.019 b	2.668 ± 0.004 i	3.446 ± 0.015 d	2.102 ± 0.010 k	2.772 ± 0.015 h	3.743 ± 0.006 c	2.991 ± 0.003 f	7.223 ± 0.035 a	3.064 ± 0.017 e	2.899 ± 0.018 g	1.577 ± 0.040 m	2.260 ± 0.011	1.941 ± 0.029 l
**Ser**	0.837 ± 0.002 e	0.646 ± 0.001 i	0.795 ± 0.004 f	0.530 ± 0.002 j	0.848 ± 0.003 e	0.709 ± 0.004 h	0.889 ± 0.001 c	1.204 ± 0.005 a	0.871 ± 0.002 d	0.930 ± 0.003 b	0.647 ± 0.025 i	0.698 ± 0.002 h	0.741 ± 0.004 g
**Thr**	0.238 ± 0.000 g	0.202 ± 0.001 i	0.227 ± 0.001 h	0.162 ± 0.000 j	0.209 ± 0.001 i	0.306 ± 0.001 f	0.312 ± 0.001 f	0.628 ± 0.003 a	0.403 ± 0.002 c	0.517 ± 0.002 b	0.352 ± 0.013 e	0.385 ± 0.001 d	0.409 ± 0.003 c
**Tyr**	0.020 ± 0.000 i	0.028 ± 0.000 f	0.025 ± 0.001 gh	0.017 ± 0.000 j	0.023 ± 0.000 h	0.048 ± 0.000 c	0.036 ± 0.000 d	0.074 ± 0.000 a	0.027 ± 0.002 f	0.026 ± 0.000 fg	0.033 ± 0.002 e	0.037 ± 0.001 d	0.069 ± 0.001 b
**Val**	0.252 ± 0.000 g	0.242 ± 0.002 h	0.262 ± 0.001 f	0.195 ± 0.001 i	0.236 ± 0.002 h	0.325 ± 0.001 e	0.322 ± 0.001 e	0.559 ± 0.005 a	0.341 ± 0.002 d	0.426 ± 0.001 b	0.346 ± 0.010 d	0.347 ± 0.002 d	0.365 ± 0.003 c
**Orn**	0.000 ± 0.000 d	0.000 ± 0.000 d	0.000 ± 0.000 d	0.000 ± 0.000 d	0.000 ± 0.000 d	0.000 ± 0.000 d	0.000 ± 0.000 d	0.036 ± 0.000 a	0.000 ± 0.000 d	0.021 ± 0.000 b	0.000 ± 0.000 d	0.000 ± 0.000 d	0.005 ± 0.001 c
**Sum**	20.970 ± 0.091 b	12.636 ± 0.054 h	15.844 ± 0.166 f	9.263 ± 0.058 k	11.158 ± 0.072 j	6.660 ± 0.140 e	14.452 ± 0.031 g	32.592 ± 0.372 a	15.892 ± 0.042 f	16.999 ± 0.125 d	12.071 ± 0.434 i	14.600 ± 0.126 g	17.541 ± 0.076 c

z Data are presented as mean ± SE (n = 3).

y Different letters indicate significant differences for each amino acid between CT and AG according to Duncan’s *post hoc* test (*P <* 0.05).

### 3.2 Imputing the Pro biosynthesis pathway and gene features

**Figure 3:**
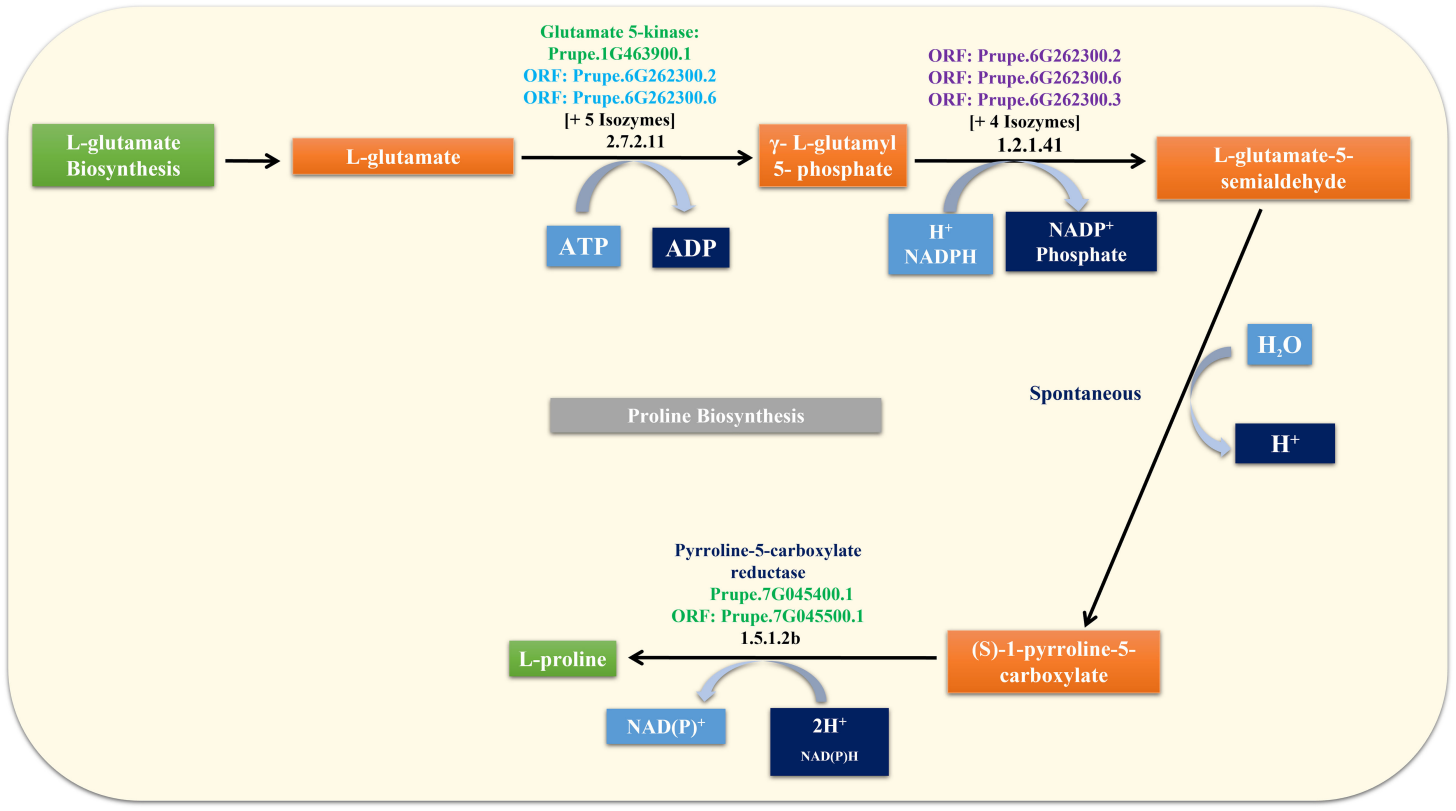
Pro biosynthesis pathway and their corresponding Pro-related genes.

The Pro molecule was used as a query in the GDRCyc database to obtain Pro-related genes and their biosynthesis pathways (**Figure 3**). Seven Pro-related genes were identified, and their details are given in **Supplementary Table 2**. Transcript and protein sequences were retrieved using the gene and transcript tools available in the GDR database. In addition, seven Pro-related genes and their attributes, such as nucleotide and amino acid length, chromosome number, isoelectric point, and molecular weight, were imputed and are listed in **Supplementary Table 2**.

### 3.3 Gene structure and *cis*-regulatory elements of Pro genes

**Figure 4:**
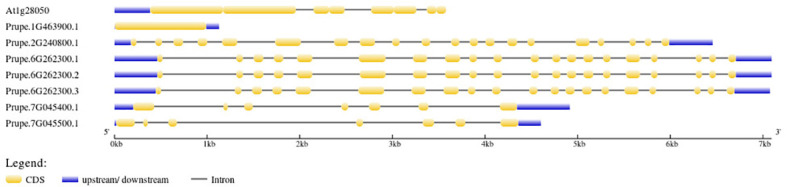
Pro-related genes organization. Orange boxes represent exons. Blue lines indicate the UTR regions. The scale bar denotes the size of the gene.

Intron and exon positions within the seven Pro-related genes were predicted, and gene structure analysis revealed the arrangement of the introns and exons (**Figure 4**). To explore the Pro-related genes and their involvement in plant growth and development, including flowering responses and stress-related processes, their promoter signal sequences were analyzed using PLACE. All *cis*-regulatory elements were imputed in the promoter regions of Pro-related genes and are given in **Supplementary Table 3**. Some of the elements were present in all Pro-related genes; hence, the other elements were unique to a few genes. EBOXBNNAPA (transcription factor (TF) target binding sites MYB, ABRE, and bHLH, seed storage protein, multidimensional cell growth), CACGTG (ABRE binding site), MYCCONSENSUSAT (drought, ABA (abscisic acid), cold, MYC recognition site), CPBCSPOR (chloroplast enhanced protein binding, cytokinin), MYB2CONSENSUSAT (MYB TF binding site, response to ABRE, drought, leaf, and seed), WRKY71OS, WBBOXPCWRKY1, WBOXATNPR1, WBOXNTERF3, and WBOXNTCHN48 (WRKY TF binding site, W box elements), DRECRTCOREAT (DRE/CRT (dehydration-responsive element/C-repeats), RAV1AAT (leaf, shoot, root specific element), CCAATBOX1 (regulation of flowering), LTRECOREATCOR15, LTRE1HVBLT49 (ABA, drought, low temperature responsive element, leaf, shoot), ASF1MOTIFCAMV (MeJA-responsive elements), SORLIP1AT (seed, root, phytochrome A gene expression, and light specific element), POLLEN1LELAT52 (pollen specific gene activation), CIACADIANLELHC (circadian expression element), SURECOREATSULTR11 (root specific expression), PYRIMIDINEBOXOSRAMY1A (sugar responsive elements) were imputed in direct upstream regions of seven Pro-related genes (**Supplementary Table 3**). Furthermore, few *cis*-regulatory elements were present in only one Pro-related gene, including UP2ATMSD (involved in growth; Prupe.1G463900.1), XYLAT (xylem specific element; Prupe.1G463900.1), POLASIG3 (response to plant polyadenylation signal; Prupe.7G045500.1), and GARE1OSREP1 (GA-responsive element; Prupe.2G240800.1) (**Supplementary Table 3**).

### 3.4 Functional GO annotation

**Figure 5:**
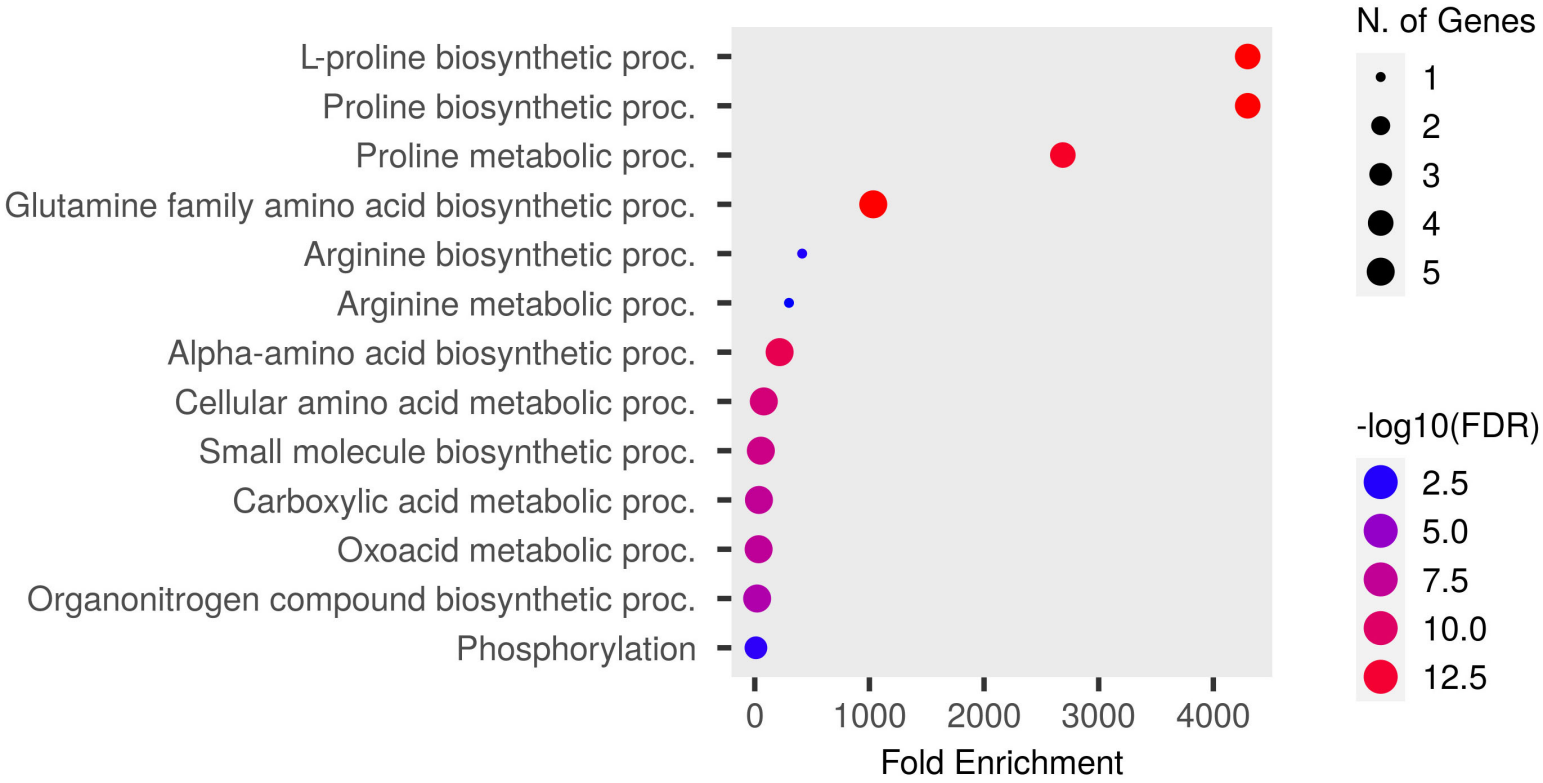
Dot plot of Pro related genes and their GO-enriched biological processes. Pro-producing genes falling in each fold enrichment GO biological process term is directly proportional to the count ball size. The balls are color-shaded according to the significant enrichment threshold level (−log10FDR) FDR *P*-value (≤ 0.05) for Pro genes from the ShinyGO *v*0.741 online tool.

**Figure 6:**
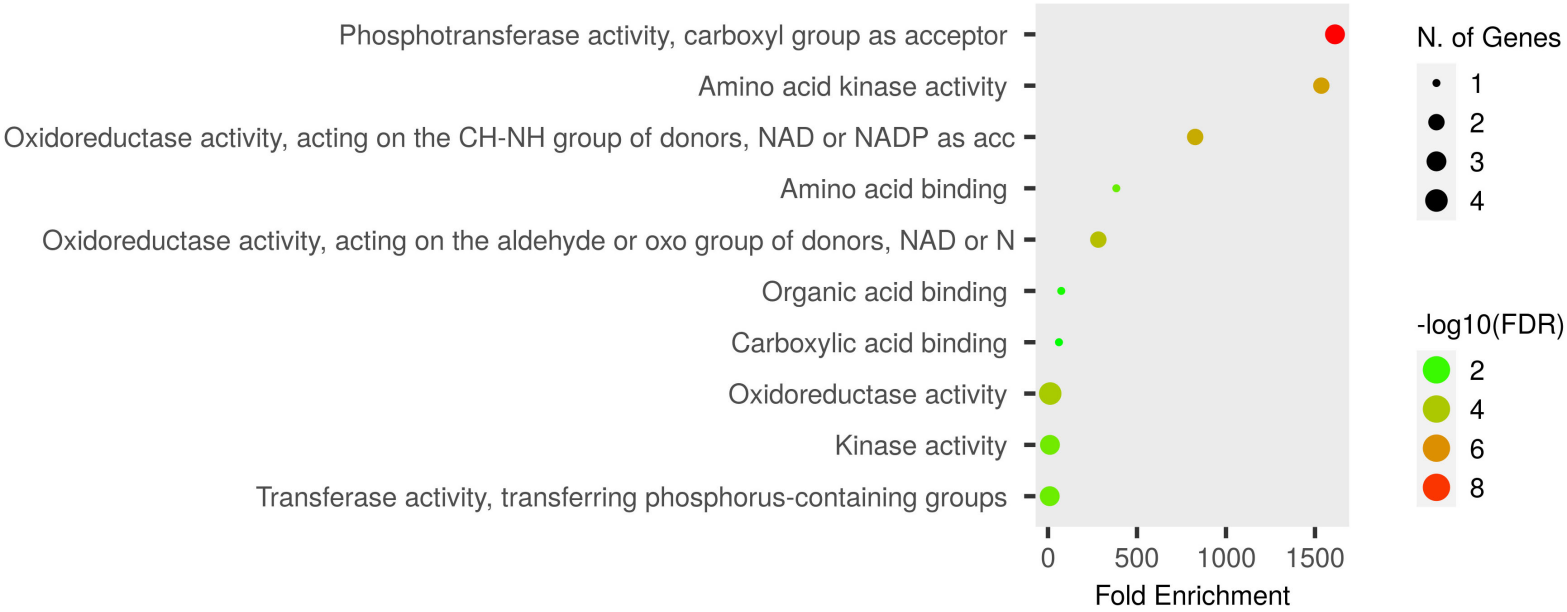
Dot plot of Pro-related genes and their associated GO-enriched molecular functions. Pro-related genes falling in each fold enrichment GO molecular function term is directly proportional to ball size count. The balls are color-shaded according to the significant enrichment threshold level (−log10FDR) FDR *P*-value at ≤ 0.05 for Pro genes from the ShinyGO *v*0.741 online tool.

Functional GO enrichment was conducted using an inbuilt hypergeometric test, which identified fold-enriched GO terms for Pro-related genes. These genes are significantly involved in various biological and molecular processes. The predicted biological processes included proline biosynthesis and metabolism, glutamine and arginine biosynthesis, cellular amino acid synthesis, phosphorylation, and other metabolic processes (**Figure 5**). The molecular functions of these genes correspond to various catalytic activities such as oxidoreductase and kinase activities, phosphotransferase activity, and carboxylic acid-binding activity, etc. (**Figure 6**).

### 3.5 Gene synteny

**Figure 7:**
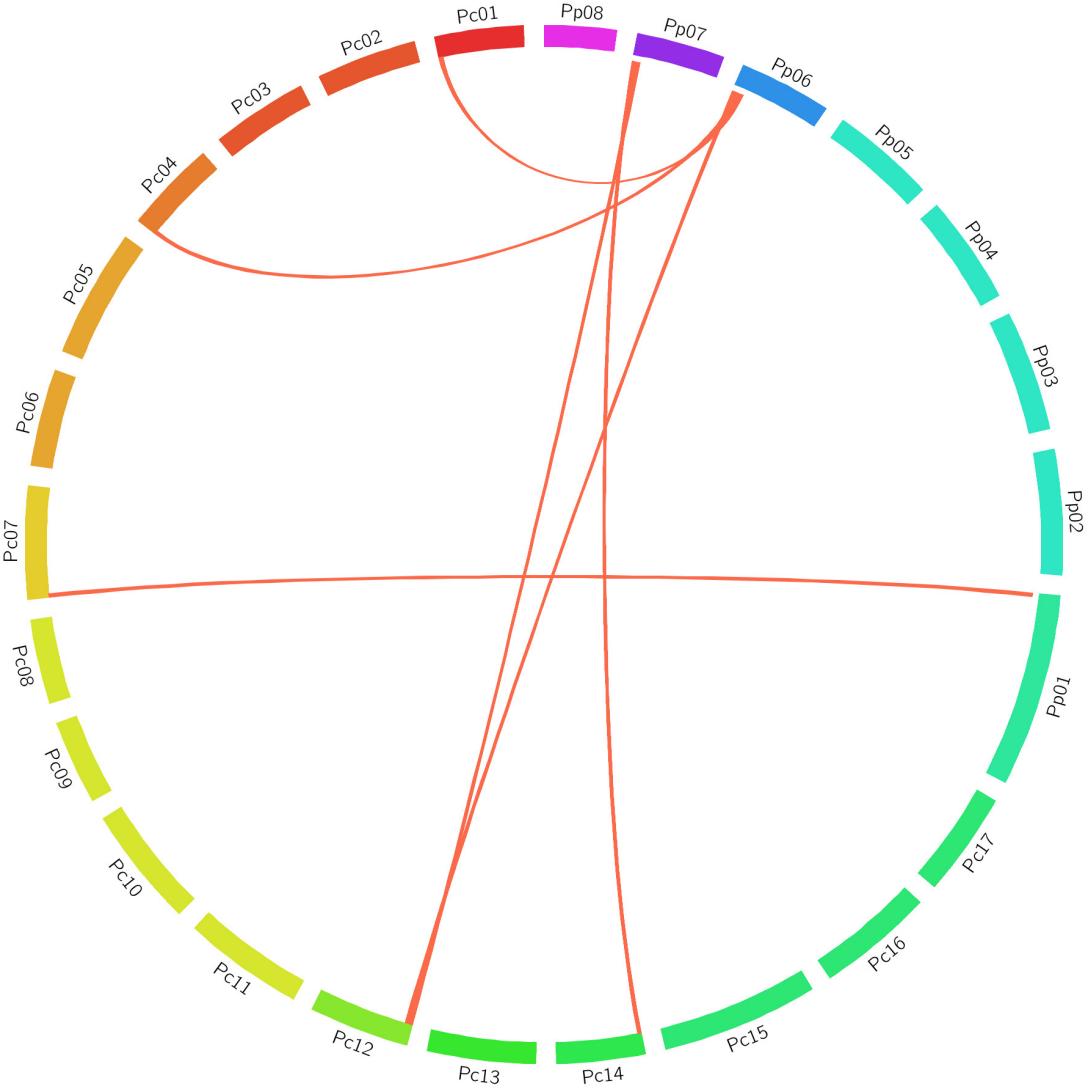
Chromosomal relationship of Pro-related genes between *P. persica* and *P. communis*. All segments are designated as chromosomes, and the synteny of genomic regions is marked in red.

Cross-species comparison in GDRCyc revealed an orthologous relationship between Pro-related genes from *P. persica* and *P. communis* in accordance with the gene location in contrast to their corresponding chromosomes. The gene syntenic relationship revealed the maximum relationship between *P. persica* and *P. communis* [six Pro-related genes (86%)] (**Figure 7**; **Supplementary Table 4**).

### 3.6 Transcriptional expression profiling

**Figure 8:**
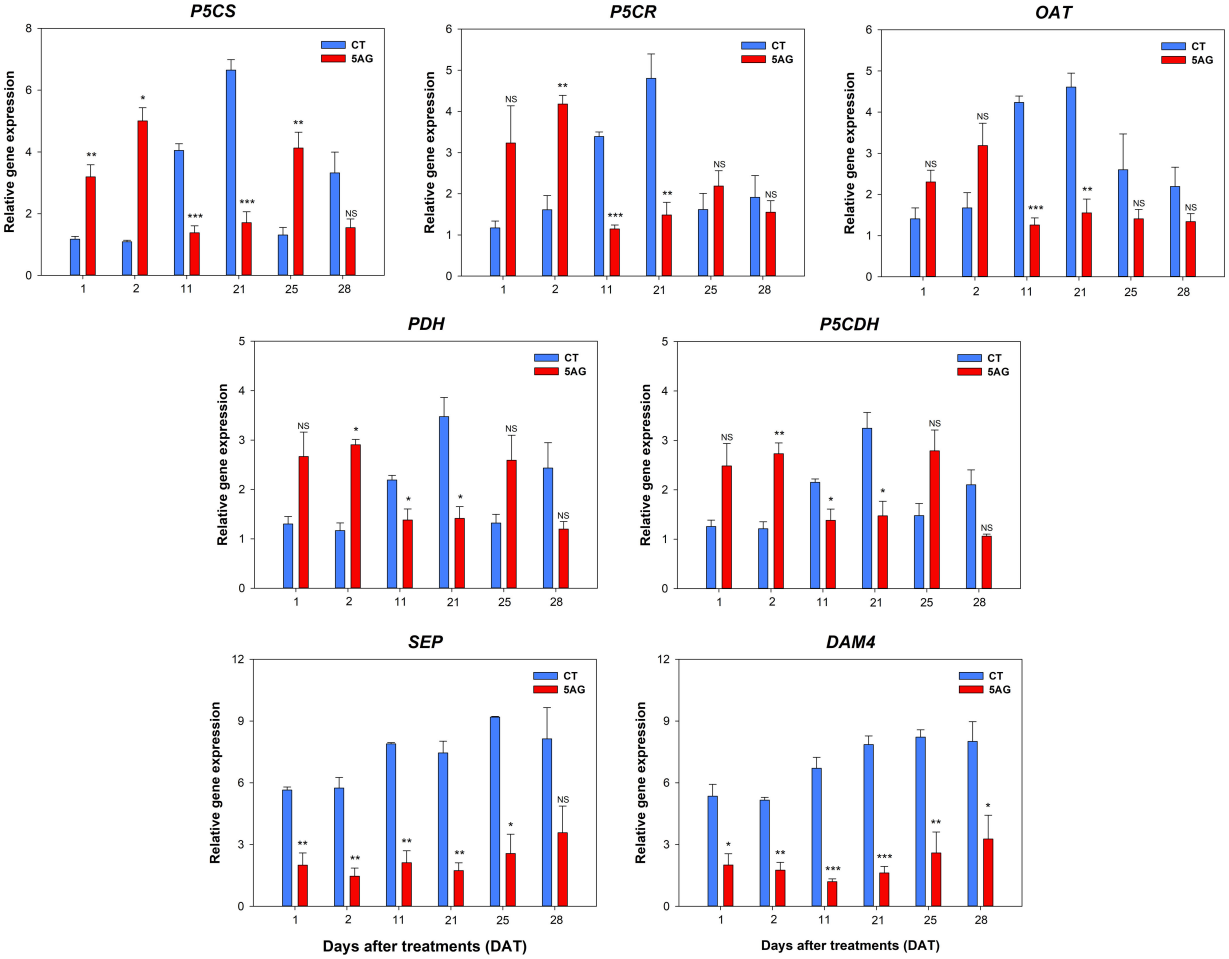
Relative expression values of Pro-related genes and flowering responsible genes extracted from control (CT) and 5AG-treated (5AG) peach shoot samples collected at different time points (1, 2, 11, 21, 25, and 28 DAT) were analyzed by qRT-PCR and presented as bar diagrams. Pro-related genes: *P5CS*, *P5CR*, *OAT*, *PDH*, and *P5CDH*; flowering responsible genes: *SEP* and *DAM4*. *RPII* was used as an endogenous control to normalize the data. Each value in the bar diagram represents the mean of three independent replicates (n = 3) and the error bar represents the standard error of mean. The presence of asterisks indicates a statistically significant difference compared to CT. *P < 0.05, **P < 0.01, ***P < 0.001, NS indicates non-significant.

The expression of Pro pathway-associated genes (*P5CS*, *Δ^1^*-pyrroline-carboxylate synthetase; *P5CR*, P5C reductase; *OAT*, ornithine *δ*-aminotransferase; *PDH*, proline dehydrogenase; and *P5CDH*, P5C dehydrogenase) and flowering-responsive genes (*DAM4*, dormancy-associated MADS-box 4 and *SEP*, SEPALLATA) were profiled using qRT-PCR in both CT and 5AG treatments at 1, 2, 11, 21, 25, and 28 DAT. The relative fold-change in gene expression showed that all Pro pathway genes were differentially expressed between CT and 5AG (**Figure 8**). Interestingly, the expression of Pro pathway-associated genes displayed a constant and similar pattern regardless of anabolic (*P5CS* and *P5CR*) and catabolic (*PDH* and *P5CDH*) processes. Pro pathway-associated genes in 5AG were upregulated at 1 to 2 DAT compared to CT, whereas they showed low expression levels from 11 to 28 DAT. In contrast, the expression of these genes in the CT gradually increased from 1 to 11 DAT and reached its highest expression at 21 DAT. Simultaneously, the expression of these genes showed the greatest differences (2.2 to 3.9 times) between CT and 5AG at 21 DAT. Among the Pro pathway-associated genes, *P5CS*, which converts Glu to P5C/GSA during Pro anabolism, was the most highly expressed. In CT, the expression levels of *P5CS*, *P5CR*, and *OAT*, which are directly related to Pro biosynthesis, were comparatively higher than those of *PDH* and *P5CDH* at 21 DAT, whereas all Pro pathway-associated genes in 5AG retained moderate expression levels (approximately 1.4 to 1.7). The *OAT* gene, which is involved in the ornithine pathway of Pro synthesis, was also activated during the peach flowering period (all DATs) in both CT and 5AG plants. Notably, *DAM4* and *SEP* exhibited substantial differences during flowering. For all DATs, these two genes were regulated 2.3 to 4.8 times higher in CT than in 5AG. Altogether, these results prove that 5AG leads to flowering delay by regulating the expression of both Pro pathway-associated and flowering genes. The obtained results also indicate their involvement in the further functional characterization of Pro in flowering delay mechanisms.

## 4 Discussion

Owing to climatic change, average air temperatures in the southern region of South Korea have increased by more than ~3 °C over the past decade, leading to early spring and advanced full blooming of fruit trees. In particular, the average full blooming date of ‘Kawanakajima Hakuto’ and other Rosaceae deciduous fruit trees has advanced by 12 days (Park and Shin, 2022). These increased temperatures trigger DA after the chilling requirements are fulfilled (Saxe et al., 2001), increasing the risk of frost damage owing to premature/early DA in spring.

Park and Shin (2022) reported that treatment with 5AG was the most effective at delaying peach blooming by up to 9 days compared to CT. Building upon our previous reports, the present study investigated the amino acid content and its role in flowering delay in CT- and 5AG-treated shoots of peach plants at different time intervals (1, 2, 11, 21, 25, and 28 DAT). Our results revealed specific differences in the changes in three amino acids (Arg, Glu, and Pro) between 5AG and CT during peach flower bud development. In CT, the levels of Arg, Glu, and Pro amino acids were elevated by 4.6-, 1.2-, and 2.4-fold compared to 5AG in 21 DAT, respectively, when the phenological stage of flower buds reached the 5th stage (first pink) (**Figure 2**). However, the content of the three amino acids in 5AG, which was only in the 4th stage (calyx red), was significantly lower than that in CT. Indeed, the three amino acids (Arg, Glu, and Pro) and their biosynthetic pathways are interconnected (Kanehisa and Goto, 2000) (**Supplementary Figure 3**). In particular, Pro is easily interlinked and interconverted with Glu and Arg using *Δ^1^*-pyrroline-5-carboxylate (P5C). Arg and Glu are considered secondary metabolites, whereas Pro acts as an efficient signaling and precursor molecule for the synthesis of other amino acids and secondary metabolites (Szabados and Savouré, 2010; Hildebrandt et al., 2015). Furthermore, Pro biosynthesis is independently controlled by the Glu → Orn → Arg pathway, and enhanced production of Pro from absorbed nitrogen may be partially compensated by reduced Glu levels due to increased flux toward Orn/Arg and Pro (Majumdar et al., 2016). Based on these results, we hypothesized that Pro metabolism plays an important role in the flowering delay following 5AG treatment.

The adverse climatic conditions faced by plants can alter their amino acid metabolism. Pro metabolism helps restore and/or maintain cellular homeostasis. Pro acts as a compatible osmolyte, cellular ROS balance regulator, chemical mediator of chaperones, and response to various biotic and abiotic stresses. It is also involved in nitrogen fixation and seedling development, particularly in the division of shoots and root tips. Pro activates floral shoot apical meristems for flower development and enhances the activity of various enzymes (Deuschle et al., 2004; Kavi Kishor and Sreenivasulu, 2014; Kavi Kishor et al., 2015). Pro also controls the dynamics of numerous genes and significantly influences overall plant growth and development (Szabados and Savouré, 2010; Hildebrandt et al., 2015; Shin et al., 2018; Wei et al., 2022). Pro is synthesized from Glu (cytosol and plastids) or ornithine (Orn) (mitochondria) via a two-step enzymatic reaction that shares the intermediate P5C (**Supplementary Figure 4**) (Szabados and Savouré, 2010; Verslues and Sharma, 2010; Wang et al., 2015). The enzymes used were P5CS and OAT. P5CS and P5CR convert Glu into Pro (Glu → P5C/Glu-semialdehyde (GSA) → Pro). In contrast, PDH catabolizes Pro to P5C and P5CDH converts the tautomeric form of P5C (GSA) into Glu (Pro → P5C/GSA → Glu) (Funck et al., 2012). Pro metabolism is controlled by these enzymes. Generally, P5CS, OAT, and PDH control the transcriptional regulation, while P5CR and P5CDH are involved in both transcriptional and post-transcriptional processes (Giberti et al., 2014). In addition, in Pro metabolism, *P5CS*, *P5CR*, and *OAT* genes are related to biosynthesis, whereas *P5CDH* and *PDH* genes are associated with catabolism (Wei et al., 2022). Pro biosynthesis is activated, and its catabolism is repressed under stress conditions. When ideal conditions are reestablished, the opposite regulation is triggered (Xue et al., 2009). Furthermore, the accumulation of Pro may be related to the degree of tolerance, and the positive correlation of Pro accumulation depends on the species and their adaptation to stress and normal physiological conditions (Hien et al., 2003; Mattioli et al., 2009; Xue et al., 2009; Wei et al., 2022). However, to the best of our knowledge, there is still a lack of understanding of Pro metabolism, particularly in relation to flowering development and delay mechanisms. Hence, this study focused on Pro metabolism to identify flowering delay mechanisms by understanding the molecular, physiological, and physiochemical properties during the flowering delay process.

Analysis of *cis*-regulatory elements in the Pro-related gene promoter sequences revealed that these members are involved in different molecular physiological and stress responses. Some elements showed multiple types of flowering, pollen, light, roots, seeds, shoots, and abiotic stress. Defense signaling hormones, such as salicylic acid and jasmonic acid, show the involvement of Pro-related genes in different signaling pathways (Filichkin et al., 2004; Wenkel et al., 2006; Deb and Kundu, 2015; Bi et al., 2016; Zhang et al., 2016; Muthuramalingam et al., 2020). Many *cis*-regulatory elements have been found to directly target factors, such as ABRE, DRE/CRT, MYB, MYC, bHLH, and WRKY TFs (Narusaka et al., 2003; Yamaguchi-Shinozaki and Shinozaki, 2005; Hou et al., 2012; Deb and Kundu, 2015). Interestingly, we found that all the Pro-related genes possessed specific elements related to flowering, pollen, shoot, and root, suggesting their potential role in flowering delay in 5AG-treated plants. Further studies are required to unveil the function of Pro-related genes in flowering delay and the other biological functions of these genes.

Chromosomal mapping of Pro-related genes and their encoding proteins in *P. persica* and *P. communis* was predicted to delineate the gene synteny between these plants. *P. persica* Pro-related genes showed maximum collinearity (86%) with *P. communis* Pro-associated genes, and these results revealed molecular insights about the Rosaceae family. Similarly, a close syntenic relationship has also been observed between *PpGRF* and *PpNAC* TFs (Zhuo et al., 2018; Liu et al., 2022). Chromosomal mapping information will pave the way for analyzing the evolutionary process of Pro-related genes and the study of crucial molecular genetic traits in the Rosaceae family. Furthermore, the study also suggests that 5AG treatment may also play a pivotal role in the flowering delay of other Rosaceae family plants.

qRT-PCR analysis of Pro pathway genes (*P5CS*, *P5CR*, *OAT*, *PDH*, and *P5CDH*) showed that they were differentially expressed in CT- and 5AG-treated peach shoot samples. The expression of all genes in CT gradually increased from 1 DAT and reached its highest expression level at 21 DAT, when Pro content was also the highest. Although all genes associated with the Glu and Orn pathways in 5AG showed higher expression than those in CT in the early stage (1 to 2 DAT), their expression was significantly suppressed from 11 to 21 DAT. In particular, we noted differences in the relative fold-changes of these genes and the amino acid content between 5AG and CT at 21 DAT. At this time, when CT reached in 5th stage, all Pro pathway-associated genes, including anabolism-related genes (*P5CS*, *P5CR*, and *OAT*) and catabolism-related genes (*PDH*, and *P5CDH*) were expressed significantly higher in CT than in 5AG, among them, *P5CS* was highest. In addition, genes *P5CS*, *PDH*, and *P5CDH* induce the development of reproductive organs (Deuschle et al., 2004; Rizzi et al., 2015). Similarly, high expression levels of the *P5CS*, *P5CR*, and *PDH* genes were observed during the development of meristematic tissues, including the shoot apex, root tips, and inflorescences, as well as during stress responses (Rizzi et al., 2015). In particular, Wei et al. (2022) reported that *P5CS2*, *OAT*, and *PDH* are highly expressed in grapevine flowers. Furthermore, *P5CS1* overexpression led to early flowering in *Arabidopsis* (Mattioli et al., 2009). Moreover, the amino acid analysis results (**Table 1**; **Figure 2**) closely resembled the expression patterns of Pro-related genes, and the three amino acids (Arg, Glu, and Pro) also peaked at 21 DAT. In particular, our results revealed that higher expression of Pro-synthesis genes *P5CS* and *P5CR* led to increased Pro content, whereas the low expression of these genes by 5AG resulted in decreased Pro content. In particular, Pro is a multifunctional amino acid that can influence flowering date through its effects on diverse signaling pathways, including stress responses. Under normal physiological conditions, plants accumulate higher Pro content during the transition to flower initiation (Kavi Kishor et al., 2015). Indeed, plants with low Pro content exhibit late flowering compared to normal plants (Mattioli et al., 2008; Mattioli et al., 2009). These studies certainly support a correlation between flowering delay and Pro content reduction, as indicated by our results. The upregulation of *PDH* and *P5CDH* in CT led to increased Glu content at 21 DAT, whereas 5AG had the opposite effect. In addition, the patterns of relative fold-changes in gene expression were similar for anabolism-related genes (*P5CS*, *P5CR*, and *OAT*) and catabolism-related genes (*P5CDH* and *PDH*) (**Figure 8**). Our results suggest that 5AG can induce flowering delay by modulating the entire Pro metabolism, including both anabolism and catabolism.

Pro and Arg are produced from the common precursor, Glu (Kanehisa and Goto, 2000; Funck et al., 2008; Winter et al., 2015; Majumdar et al., 2016). In addition, Arg in the Orn–Pro pathway synthesizes urea and Orn via the action of arginase. In addition, Orn is a central metabolite positioned at the intersection of the following interrelated pathways containing these amino acids: Glu → Orn → Pro/Arg, Arg → Orn → Glu/Pro, and Glu → Orn → Arg (Funck et al., 2008; Majumdar et al., 2016). Therefore, the increase in Arg content may be indirectly correlated with the upregulation of *PDH*, *P5CDH*, and *OAT*. In general, only one Pro biosynthesis pathway is activated under different physiological and stress conditions (Giberti et al., 2014). However, our study proposes that both pathways (Glu to Pro and Orn to Pro) may be activated concurrently during the flowering processes in peach trees. Thus, our study revealed that Pro metabolism is strongly correlated with the CT of flowering time in the phenological development of peach flowers. Moreover, the flowering responsible genes *DAM4* (Zhu et al., 2020) and *SEP* (Pi et al., 2021), were expressed up to 4.8-fold higher in CT than in 5AG during all DATs. These results strongly suggest that 5AG treatment significantly delayed peach flowering.

Collectively, the current study revealed that Pro metabolism-related genes play a crucial role in the flowering delay of peaches through physiological and metabolic analyses. This suggests an intimate relationship between flowering delay and Pro metabolism. In view of the importance of peach flowering delay in avoiding frost damage, this study represents the first comprehensive report identifying the flowering delay mechanism induced by 5AG in *P. persica*. With the demonstrated effectiveness of sodium alginate and CaCl_2_ treatment in delaying flowering, there is a growing need for the large-scale application of flowering retardants in peach orchards. Consequently, further research is crucial to facilitate the commercialization of these treatments.

## 5 Conclusion

Recent advances in computational omics have uncovered the transcriptional regulation of Pro and metabolic responses in peach flowering. Pro metabolism has been studied in crop plants and some tree species but not in peaches. Previous studies have shown that 5AG treatment can delay flowering in peaches. Our study supports these results and suggests that 5AG treatment (at different day intervals: 1, 2, 11, 21, 25, and 28 DAT) unveiled novel avenues for delay the 2nd stage of peach floral buds to full blooming. Profiling of 19 amino acids in peach shoots revealed that 5AG-treated shoots had lower levels of Arg, Glu, and Pro than CT shoots did. Interestingly, their biosynthetic pathways are interconnected and are closely related to Pro metabolism. Considering the amino acid profiling results, our study aimed to elucidate delayed flowering metabolism by understanding the role of Pro pathway-related genes in the flowering response. Computational analyses identified Pro-metabolism related genes and their gene structure, gene ontology enrichment, gene features, gene synteny, and *cis*-acting elements, which denote diverse biological functions of these Pro metabolism-related genes, including flowering responses. Transcriptional analysis showed that elevated expression of Pro-metabolism related genes such as *P5CS* and *P5CR* led to increased Pro content, whereas low expression of these genes by 5AG treatment resulted in reduced Pro content levels. Furthermore, at the 5th stage of CT, Pro metabolism genes (*P5CS*, *P5CR*, *OAT*, *PDH*, and *P5CDH*) and their expression levels were significantly higher in CT than in 5AG. This suggests that 5AG treatment may restrain the expression of genes related to Pro accumulation, thereby controlling Pro biosynthesis. In addition, our findings revealed a direct link between Pro content reduction and delayed flowering. Notably, flowering responsible genes did not exhibit significant differences in expression between the 5AG-treated and CT samples. These results provide strong evidence that 5AG treatment significantly delays flowering by controlling Pro metabolism.

Furthermore, this study hypothesizes that Pro metabolism-related genes and their encoding genetic nature may interact with downstream genes, activating functionally associated players that may be involved in diverse biological pathways. These pathways may include flowering response pathways such as gibberellic acid, photoperiod pathway, light quality pathway, autonomous and vernalization pathways (yet to be characterized), and stimulation of flowering-related TFs. Advanced studies, including RNA-seq and integrated multi-omics approaches, will further validate the in-depth molecular insights into Pro metabolism-related genes in peach flowering delay and other flowering response pathways under 5AG treatment.

## Data availability statement

The original contributions presented in the study are included in the article/**Supplementary Material**. Further inquiries can be directed to the corresponding author.

## Author contributions

YP: Data curation, Formal analysis, Investigation, Methodology, Validation, Writing – original draft. PM: Conceptualization, Data curation, Formal analysis, Investigation, Methodology, Validation, Visualization, Writing – original draft, Writing – review & editing. JJ: Methodology, Writing – review & editing. SK: Methodology, Writing – review & editing. HS: Conceptualization, Methodology, Project administration, Resources, Supervision, Writing – review & editing.

## Supplementary material

Supplementary Figure 1 Overall framework of the present study.

Supplementary Figure 2 The five phenological stages of ‘Kawanakajima Hakuto’ peach flower. **(A)** 2nd stage = Swollen Bud; **(B)** 3rd stage = Calyx Green; **(C)** 4th stage = Calyx Red; **(D)** 5th stage = First Pink; **(E)** 6th stage = First Bloom.

Supplementary Figure 3 Arg, Glu and Pro interconnected pathways.

Supplementary Figure 4 Pro biosynthesis from Glu and Orn.
