# Supplementary figures and images for: Physiological and metabolic analyses reveal the proline-mediated flowering delay mechanism in Prunus persica
Source: Front Plant Sci. 2024 Apr 25;15:1302975. doi: 10.3389/fpls.2024.1302975 (PMC11079198; doi:10.3389/fpls.2024.1302975)

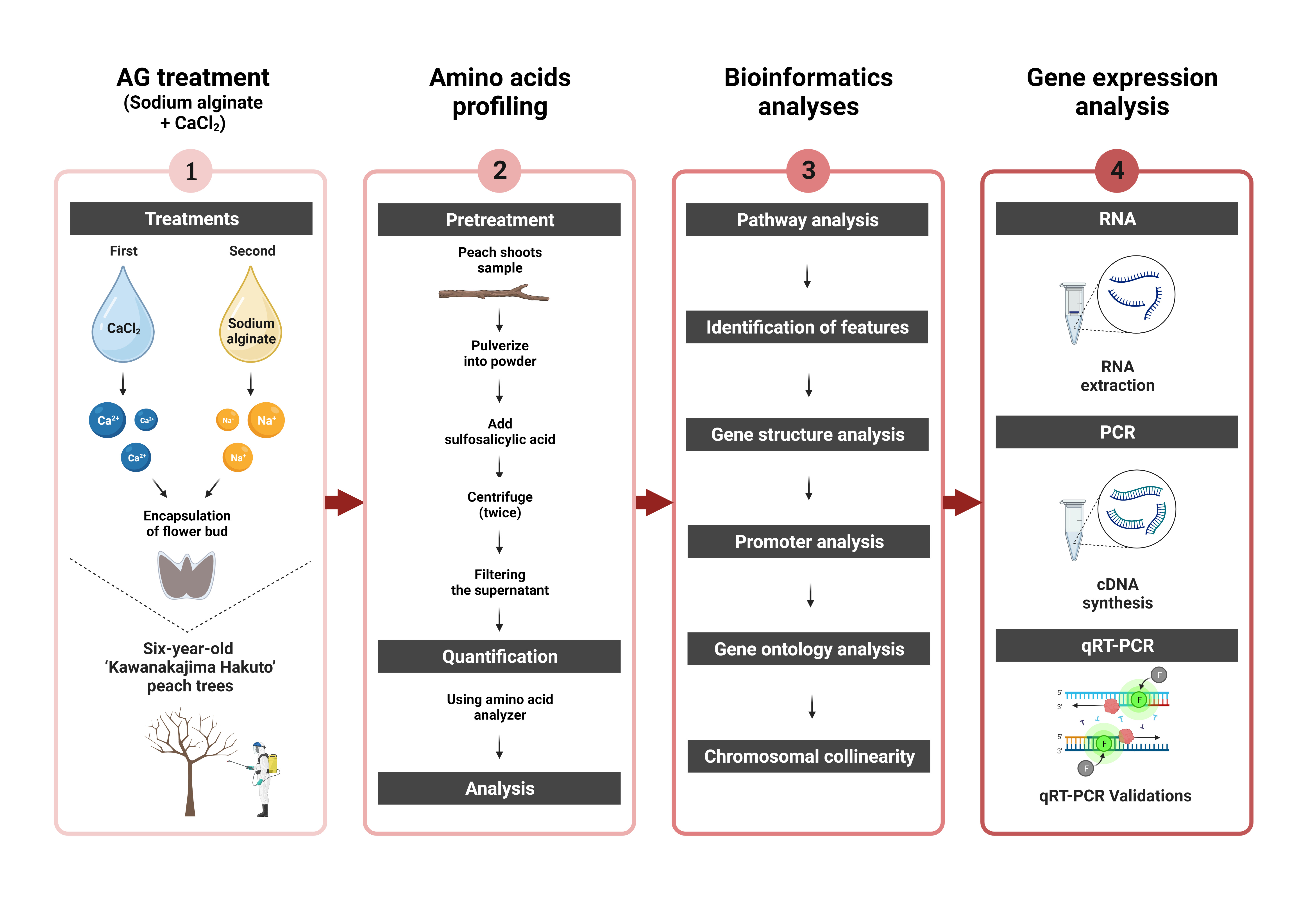

Supplement: Supplementary Figure 1 — Overall framework of the present study. [file Image_1.jpeg]

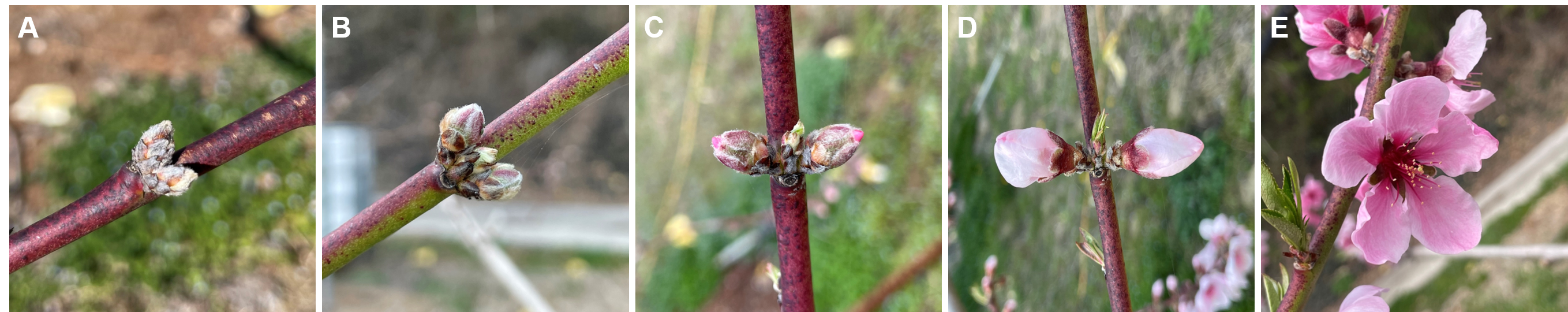

Supplement: Supplementary Figure 2 — The five phenological stages of ‘Kawanakajima Hakuto’ peach flower. (A) 2nd stage = Swollen Bud; (B) 3rd stage = Calyx Green; (C) 4th stage = Calyx Red; (D) 5th stage = First Pink; (E) 6th stage = First Bloom. [file Image_2.png]

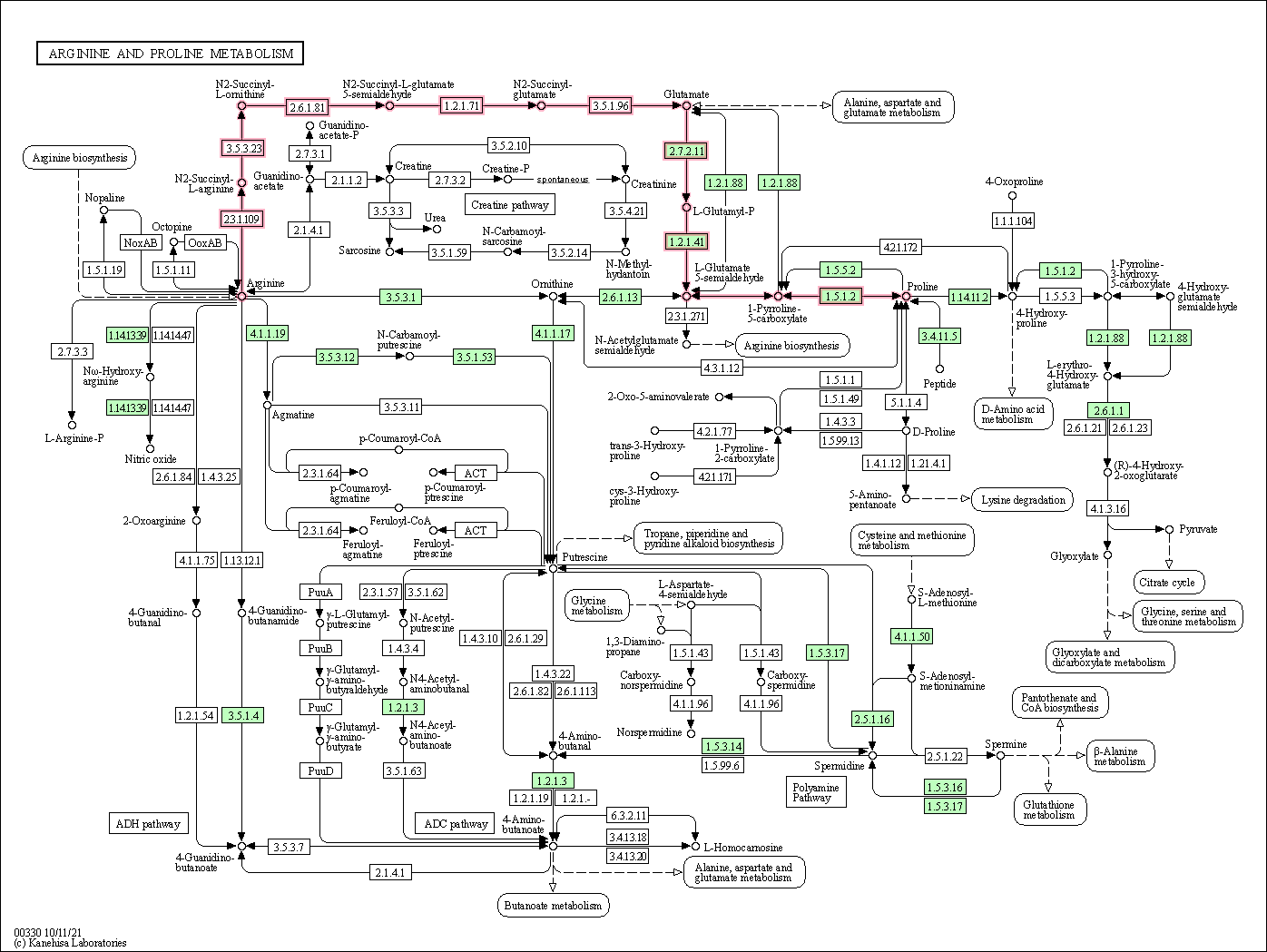

Supplement: Supplementary Figure 3 — Arg, Glu and Pro interconnected pathways. [file Image_3.png]

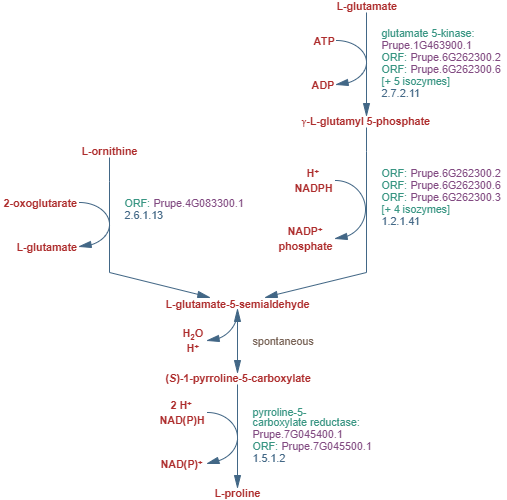

Supplement: Supplementary Figure 4 — Pro biosynthesis from Glu and Orn. [file Image_4.png]
