# Supplementary material for: Physiological and metabolic analyses reveal the proline-mediated flowering delay mechanism in Prunus persica
Source: Front Plant Sci. 2024 Apr 25;15:1302975. doi: 10.3389/fpls.2024.1302975 (PMC11079198; doi:10.3389/fpls.2024.1302975)
Supplement: Supplementary file 5 [file Table_1.doc]

**Supplementary Table1:** List of Primers used for qRT-PCR

| **Genes’** | **Forward Primer Sequence (5’-3’)** | **Reverse Primer Sequence (5’-3’)** |
| --- | --- | --- |
| ***P5CS*** | CCAAGGGGCAGCAATAAACTG | CTTCTAGGTCTTCTGCGATAA |
| ***P5CR*** | GCATCCAGGTCAGCTAAAGG | CCAGCGCTATGAAAAGGAAG |
| ***OAT*** | GTCAGAGAGCTGTGCTCAAA | ACCTCTTCCTCGAACTTCCT |
| ***P5CDH*** | TCCAAGTGCCCGAAGCATGG | GCGGACCTGATCACCAGCAA |
| ***PDH*** | GCTGCTGGCAACAAAGGCTG | TGCCACTGCCAGTTTCCCTG |
| ***RP II*** | TGAAGCATACACCTATGATGATGAAG | CTTTGACAGCACCAGTAGATTCC |
| ***DAM4*** | CGCCAGCTGAGGCAGATGAA | GCAGCTGGTGGAGGTAGCAG |
| ***SEP*** | GACACGTCGCCGTTCTTTGC | GCCACAGCAGCCAAACACAC |
