# Supplementary material for: Physiological and metabolic analyses reveal the proline-mediated flowering delay mechanism in Prunus persica
Source: Front Plant Sci. 2024 Apr 25;15:1302975. doi: 10.3389/fpls.2024.1302975 (PMC11079198; doi:10.3389/fpls.2024.1302975)
Supplement: Supplementary file 6 [file Table_2.doc]

**Supplementary Table 2:** List of Pro related genes and their attributes.

| **S. No.** | **Gene name/ ID** | **Gene size (bp)** | **Location** | **Chr. No.** | **Amino acid length** | **M. Wt.** | **pI** |
| --- | --- | --- | --- | --- | --- | --- | --- |
| 1 | Prupe.1G463900.1 | 987 | 38719089-38720075 | 1 | 328 | 34553.31 | 8.62 |
| 2 | Prupe.6G262300.2 | 6248 | 25237453-25243700 | 6 | 718 | 77476.57 | 6.28 |
| 3 | Prupe.6G262300.3 | 2157 | 25237208-25244145 | 6 | 718 | 77476.57 | 6.28 |
| 4 | Prupe.6G262300.1 | 2169 | 25237207-25244161 | 6 | 722 | 77968.18 | 6.28 |
| 5 | Prupe.2G240800.1 | 6330 | 26038666-26044996 | 2 | 717 | 77636.92 | 6.34 |
| 6 | Prupe.7G045400.1 | 4338 | 8139853-8143995 | 7 | 283 | 29675.40 | 6.72 |
| 7 | Prupe.7G045500.1 | 4338 | 8145278-8149615 | 7 | 276 | 290016.47 | 8.75 |
